# Transcriptomic landscape of human circular RNAs: unveiling molecular mechanisms in high-altitude adaptation

**DOI:** 10.3389/fgene.2026.1823611

**Published:** 2026-08-10

**Authors:** Buhe Bao, Mingxuan Yu, Wei Pang, Ruilin Wang, Wenbin Dong, Ying Bai, Rui Shi, Renjie Wang

**Affiliations:** 1 Department of Clinical Laboratory, Characteristic Medical Center of Chinese People’s Armed Police Force, Tianjin, China; 2 Department of Prevention and Therapy of Cardiovascular Diseases in Alpine Environment of Plateau, Characteristic Medical Center of Chinese People’s Armed Police Force, Tianjin, China

**Keywords:** argonaute 2 protein, circular RNAs, eukaryotic translation initiation factor 4A3, high altitude, high-altitude adaptation, hypoxia-inducible factor-1

## Abstract

High altitude (HA) is associated with environmental stress and low oxygen levels, and HA adaptation varies among populations. This study aimed to explore HA adaptation-related circular RNAs (circRNAs) and their transcriptomic profiles. We performed high-throughput analysis on male individuals from three populations at different altitudes and identified gene copy number variations (CNVs) and numerous differentially expressed circRNAs (DEcircRNAs) via pairwise comparisons. Gene Ontology (GO) and Kyoto Encyclopedia of Genes and Genomes (KEGG) analyses showed that the parent genes of DEcircRNAs were enriched in biological regulation and the hypoxia-inducible factor-1 (HIF-1) signaling pathway. Five key DEcircRNAs (hsa_circ_0044526, hsa_circ_0022498, hsa_circ_0044534, hsa_circ_0044520, and hsa_circ_0026102) interacting with RBPs (argonaute 2 protein (AGO2) and eukaryotic translation initiation factor 4A3 (EIF4A3)) were identified. Their expression increased with altitude, with the highest expression observed at 3,000 m). Overexpression of HIF-1 was positively correlated with the upregulated expression of the five circRNAs in human endothelial cells, offering preliminary correlative evidence for investigating hypoxia-associated transcriptional links underlying high-altitude adaptation. Collectively, these DEcircRNAs may contribute to HIF-1-mediated HA adaptive metabolism.

## Introduction

High-altitude (HA) regions are increasingly visited by diverse populations for various purposes, including recreation and pilgrimage (Han et al., 2024). Successful acclimatization requires coordinated physiological regulation at the cellular, tissue, and organismal levels (Ortiz et al., 2021; Xu et al., 2022; Guo et al., 2026). Since population background, geographic origin, and evolutionary history significantly dictate HA-related responses (Ferraretti et al., 2025; Seifu et al., 2024; Getu et al., 2025), understanding the population-specific nuances of these adaptations is critical (Quan et al., 2021; Lin et al., 2023; Ferraretti et al., 2024; Li et al., 2024a). However, evidence regarding the variability of circRNA expression profiles among different populations in HA environments remains limited (Ge et al., 2021; Li et al., 2024b).

Circular RNAs (circRNAs) are a class of covalently closed-loop non-coding RNAs characterized by the absence of 5′caps and 3′poly(A) tails (Hansen et al., 2013; Kos et al., 1986; Wang et al., 2014). While primarily derived from exons, circRNAs can also originate from intronic sequences (Wang et al., 2014). Functionally, many circRNAs act as competitive endogenous RNAs (ceRNAs) or miRNA sponges; by sequestering specific microRNAs (miRNAs), they alleviate the post-transcriptional repression of target genes, thereby promoting their expression (Hansen et al., 2013). Recently, circRNAs have emerged as essential mediators of metabolic regulation and promising biomarkers for diverse pathologies, including cancer (Chen et al., 2023; Yu et al., 2022; Zhu et al., 2023). Notably, circRNAs are also critical to hypoxic adaptation. For instance, research on Tibetan chickens identified 93 differentially expressed circRNAs under hypoxic conditions, with the circBRD1/novel_miR-589/APOA1 axis specifically modulating angiogenesis and embryonic hypoxic tolerance (Chen et al., 2023). Similarly, in patients with high-altitude pulmonary edema (HAPE), 200 differentially expressed circRNAs were identified, among which hsa_circ_0058497, hsa_circ_0081006, and hsa_circ_0083220 showed significant correlations with clinical indices, highlighting their potential as diagnostic biomarkers (Li et al., 2024b).

In recent years, circRNAs have been considered key biomarkers for many human diseases, including cancer, due to their important roles in metabolism. However, whether circRNAs participate in human HA adaptation and regulation remains unclear. Advanced microarray technologies have facilitated experimental approaches, enabling detection of the expression of thousands of genes (Yu et al., 2022; Zhu et al., 2023). Microarray studies using genes from HAPC patients showed that LRRC18 and HCAR3 exhibit significant expression changes following early exposure to the Qinghai–Tibet Plateau, and these two genes may serve as hub genes in the occurrence and development of high-altitude-associated polycythemia (HAPC) (Wang et al., 2022). Genomic sequencing results from Tibetan patients with HAPC indicate that EGLN3/PHD3 and PPP1R2P1 may be associated with HAPC susceptibility (Gesang et al., 2019). Thus, microarray-based genome-wide expression profiling provides an ideal method for investigating HA adaptation.

To explore population-specific transcriptomic variability, we conducted a comprehensive gene expression profiling analysis using RNA samples from male participants across three cohorts: Tianjin (0 m), Ningxia Yinchuan (1,000 m), and Xizang Linzhi (3,000 m). Differential expression analysis identified numerous circRNAs in pairwise comparisons between these populations. Gene Ontology (GO) enrichment analysis revealed that the parent genes of these differentially expressed circRNAs were primarily involved in biological regulatory processes. Furthermore, Kyoto Encyclopedia of Genes and Genomes (KEGG) pathway analysis indicated that these parent genes were significantly enriched in the hypoxia-inducible factor-1 (HIF-1) signaling pathway, among others. Notably, five circRNAs were consistently identified as differentially expressed across all three groups. Subsequent analysis of the predicted RNA-binding proteins for these candidates highlighted key interactions with proteins such as AGO2 and EIF4A3.

To validate the differential expression of the five identified candidate circRNAs, quantitative real-time PCR (qRT-PCR) was performed. The results revealed a progressive increase in the relative expression of these circRNAs across altitudes, with the highest levels observed in the 3,000 m group. Furthermore, overexpression of HIF-1 in human endothelial cells significantly upregulated the expression of these circRNAs compared to the control group. In conclusion, we identified five consistently differentially expressed circRNAs (hsa_circ_0044526, hsa_circ_0022498, hsa_circ_0044534, hsa_circ_0044520, and hsa_circ_0026102) across populations at varying altitudes, demonstrating their potential role in HIF-1-mediated high-altitude adaptation.

## Materials and methods

### Sample preparation

This retrospective study used de-identified residual blood samples from routine health examinations. Thirty eligible male individuals aged 18–35 years were equally divided into three groups (n = 10): Tianjin, Ningxia Yinchuan, and Xizang Linzhi cohorts. The inclusion criteria were as follows: healthy individuals with normal physical examination results and at least 3 consecutive years of local residence. The exclusion criteria were as follows: chronic or hereditary diseases, long-term medication use, irregular lifestyle, and short-term residence. Blood samples were collected from the antecubital vein between 6 and 8 a.m. (22 °C ± 2 °C) and irreversibly anonymized. To address the insufficient RNA yield for individual microarray hybridizations, we randomly pooled equal volumes (400 μL) of RNA from participants within each group to create three non-overlapping composite pools. Each pool was then analyzed as a distinct biological replicate without technical duplication.

### Total RNA extraction

Whole blood samples were collected using PAXgene Blood RNA tubes (PreAnalytiX, Switzerland; QIAGEN). Total RNA was extracted and purified using the QIAGEN serum/Plasma Kit (Cat 217184, QIAGEN) following the manufacturer’s standard instructions. RNA concentration, purity, and integrity were verified using the NanoDrop ND-2000 spectrophotometer and Agilent Bioanalyzer 2100 (Agilent Technologies, Santa Clara, CA, United States).

### Oligonucleotide microarray hybridization

Total RNA was amplified and labeled using the Agilent Low Input Quick Amp Labeling Kit, One-Color (Cat# 5190–2305). Labeled cRNA was purified using the RNeasy Mini Kit (Cat#74106, QIAGEN). Hybridization was performed in an Agilent hybridization oven at 65 °C with rotation at 10 rpm for 17 h with 1.65 μg of Cy3-labeled cRNA per array. Slides were washed using the Gene Expression Wash Buffer Kit (Cat#5188–5327, Agilent). Microarrays were scanned using an Agilent Microarray Scanner (G2565CA) at a resolution of 3 μm, with PMT set at 100%, 20-bit depth.

### Gene expression profile

Raw expression data were extracted using Agilent Feature Extraction software (version 12.0) and normalized by quantile normalization using the limma package in the R/Bioconductor platform. Quality control analyses were conducted, including principal component analysis (PCA), sample clustering analysis, and sample correlation analysis. All samples were processed in a single batch to avoid batch effects, and three independent pooled RNA biological replicates were included for each group (sea level (0 m), 1,000 m, and 3,000 m). All raw array signals were log-transformed to satisfy normality assumptions for a Student’s t-test and ANOVA. The Benjamini–Hochberg (BH) procedure was applied to calculate the false discovery rate (FDR) for multiple-testing correction. Differentially expressed circRNAs were defined as transcripts with |log_2_ (fold change)| > 1 and FDR <0.05.

### Gene ontology analysis

GO enrichment was performed using Fisher’s exact test using the clusterProfiler R package (v4.20.0; https://bioconductor.org/packages/release/bioc/html/clusterProfiler.html) from the R/Bioconductor platform. Genes were assigned to three major GO categories: biological process (BP), molecular function (MF), and cellular component (CC). The thresholds for significant enrichment were set to a minimum of two differentially expressed host genes mapping to a given term and a raw *p* < 0.05. For visualization, the top 30 GO terms, ranked in descending order by enrichment factor, were displayed in dot plots. Dot size corresponds to the count of enriched DEGs, and the color gradient represents the FDR-adjusted statistical significance.

### KEGG enrichment analysis

KEGG pathway enrichment was performed using the host genes of the differentially expressed circRNAs. Fisher’s exact test was used for enrichment analysis via the clusterProfiler package in R/Bioconductor. The screening criteria for significant enrichment included a minimum of two enriched DEGs per pathway and a raw *p* < 0.05. The enrichment factor was calculated exactly as described for the GO analysis. The top 30 pathways, ranked in descending order by enrichment factor, were visualized using dot plots. KEGG pathways were classified into six major categories: cellular processes, environmental information processing, genetic information processing, human diseases, metabolism, and organismal systems. Dot size represents the number of enriched DEGs, and the color gradient reflects the FDR-adjusted statistical significance.

Cell culture: Human umbilical vein endothelial cells (HUVECs) were purchased from the Cell Bank of the Chinese Academy of Sciences (Shanghai, China). Cells were cultured in Dulbecco’s modified Eagle’s medium (DMEM; Sigma, MO, United States) supplemented with 10% fetal bovine serum (FBS; Invitrogen, CA, United States), 100 U/mL penicillin, and 100 μg/mL streptomycin (Invitrogen, CA, United States). Cells were maintained in a humidified incubator with 5% CO_2_ at 37 °C.

### Cell transfection

HIF-1α vector control vectors were designed by RiboBio (Shanghai, China). Transfections were performed using Lipofectamine 2000 (Invitrogen, CA, United States) following the manufacturer’s protocols.

### qRT-PCR validation

qRT-PCR was performed using the QuantiNova™ SYBR Green PCR Kit (QIAGEN, Hilden, Germany) according to the manufacturer’s instructions. The Applied Biosystems 7900 Real-Time PCR System was used to quantify the relative expression of circRNAs. qRT-PCR primers were custom-synthesized by GenePharma (Shanghai, China). The sequences are not publicly available but can be requested from the corresponding author.

### Statistics analysis

All experimental data were analyzed using GraphPad Prism software (version 8.0) and are presented as the mean ± standard deviation (SD). Prior to statistical analysis, data were assessed for normality and homogeneity of variance. Pairwise comparisons between groups were conducted using Student’s t-test. For all transcriptome-wide comparisons, multiple-testing correction was applied using the Benjamini–Hochberg (BH) method to control the false discovery rate (FDR). Statistical significance was defined as an FDR <0.05. Statistical significance was set at *p* < 0.05. Asterisks indicate significance levels: *p* < 0.05 (*), *p* < 0.01 (**), *p* < 0.001 (***), and *p* <0.0001 (****); ns indicates no significant difference (*p* > 0.05).

### Informed consent

This study was performed using de-identified residual health examination samples. Since no personal information could be identified, the requirement for written informed consent was waived by the Institutional Review Board.

## Results

### Data source and research strategy

**FIGURE 1:**
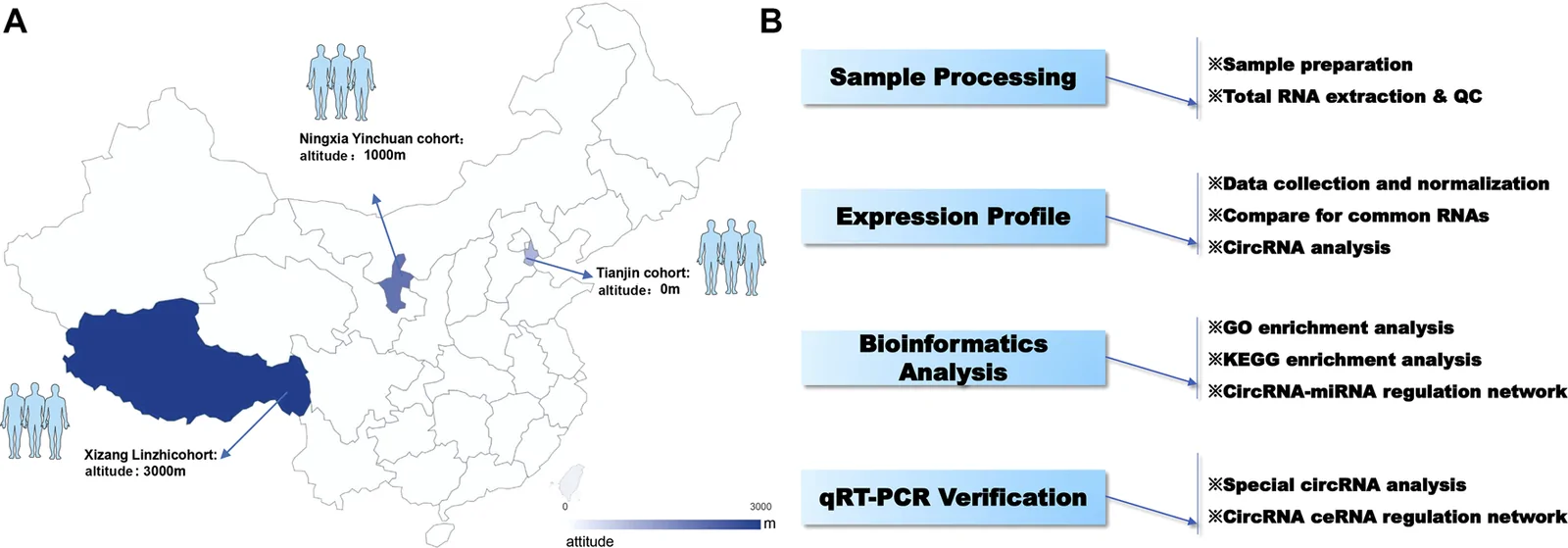
Experimental design and the research strategies. **(A)** Three population groups from different altitudes were included in this retrospective study: Tianjin cohort (sea level, 0 m), Ningxia Yinchuan cohort (1,000 m), and Xizang Linzhi cohort (3,000 m). All samples were de-identified residual health examination specimens. **(B)** Research workflow of this study. The workflow comprised four main stages: sample processing, expression profiling, bioinformatic analysis, and qRT-PCR validation. Sample processing involved sample preparation, total RNA extraction, and quality control. Expression profiling included data acquisition, normalization, comparative analysis of common RNAs, and identification of differentially expressed circRNAs. Bioinformatic analysis encompassed GO enrichment, KEGG pathway enrichment, and circRNA–miRNA regulatory analysis. qRT-PCR validation involved the quantification of candidate circRNAs and the construction of the circRNA–miRNA–mRNA (ceRNA) regulatory network.

To investigate altitude-associated differential circRNA expression, we selected three population cohorts located at varying altitudes: Tianjin (sea level, 0 m), Ningxia Yinchuan (1,000 m), and Xizang Linzhi (3,000 m) (Figure 1A). The comprehensive study workflow is illustrated in Figure 1B and comprises four main stages: sample processing, expression profiling, bioinformatic analysis, and experimental validation. Sample processing involved rigorous preparation, total RNA extraction, and quality control (QC). Expression profiling included data acquisition, normalization, and identification of differentially expressed circRNAs. Bioinformatic analysis encompassed GO and KEGG pathway enrichment analyses and circRNA–miRNA regulatory network construction. Finally, specific candidate circRNAs were validated by qRT-PCR to further elucidate the circRNA–miRNA–mRNA (ceRNA) regulatory axis.

### Copy number variation analysis

**FIGURE 2:**
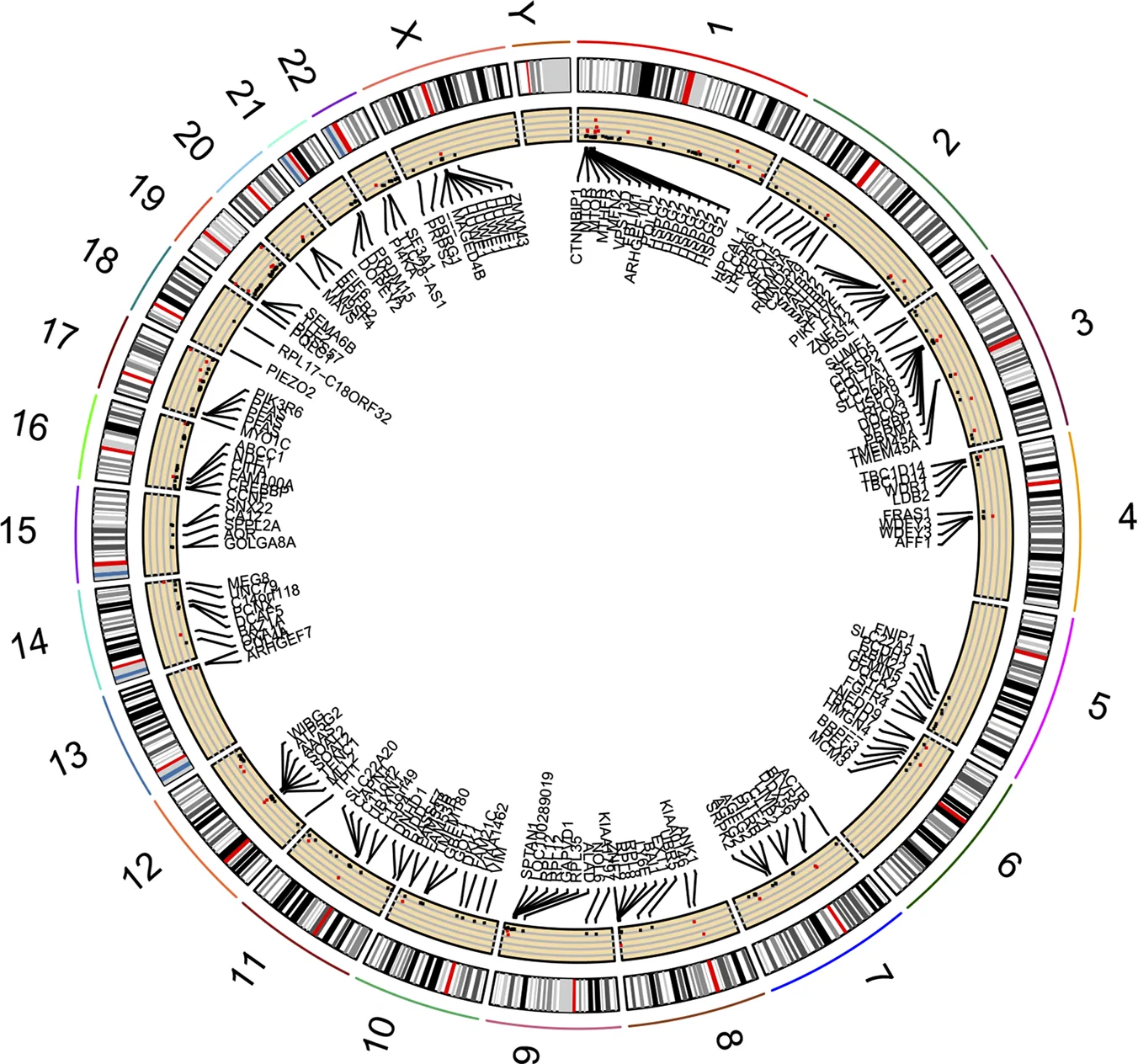
The Circos plot shows the CNVs of the human genome. The outer layer of the Circos plot represents the chromosomal structure, while the inner layer displays the genome-wide distribution of CNVs. Gene expression profiles were normalized and compared against consistently expressed transcripts. Genes harboring CNVs are illustrated in the innermost layer, where black and red dots represent copy number loss and gain, respectively.

We first analyzed the copy number variations (CNVs) of the parent genes of the identified circRNAs (Figure 2). To examine CNVs in these regions, we calculated the frequencies of copy number loss and gain across the genome. The outer layer represents the chromosomal structure, while the inner layer displays the genome-wide distribution of CNVs. Gene expression profiles were normalized and compared against consistently expressed transcripts. Genes with identified CNVs are depicted in the innermost layer, where black and red dots represent copy number losses and gains, respectively. Large-scale copy number losses were observed in specific regions, such as chromosomes 5, 15, and 18, whereas a combination of both copy number losses and gains was present in other chromosomes. No CNVs were detected on the Y chromosome.

### CircRNA expression profiles

**FIGURE 3:**
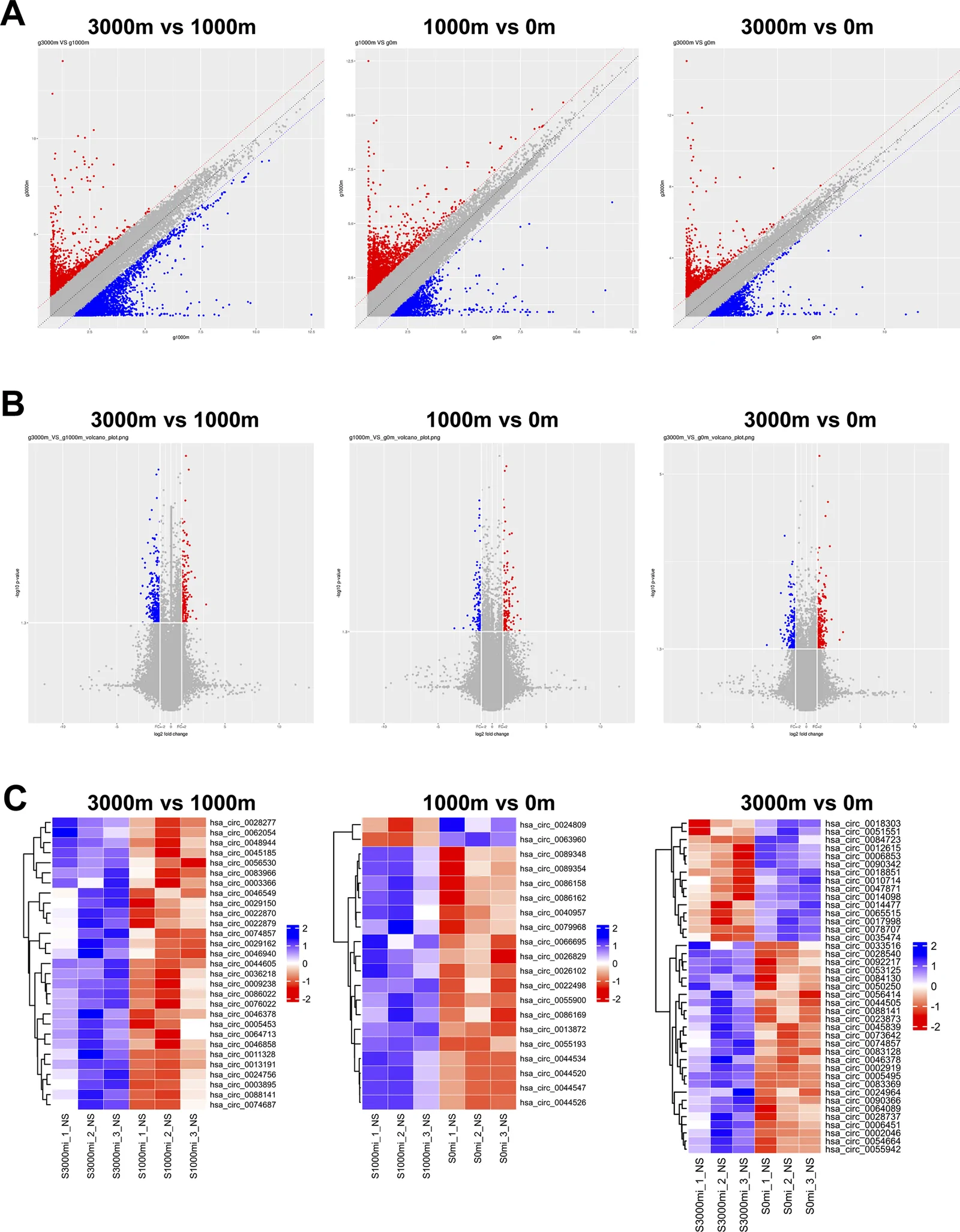
CircRNA analysis identified differentially expressed circRNAs among the three population groups. **(A)** Scatter plots showing circRNA expression differences in the 3,000 m vs. 1,000 m; 1,000 m vs. sea level (0 m); and 3,000 m vs. sea level (0 m) comparisons. Red and blue points indicate significantly dysregulated circRNAs in each comparison. **(B)** Volcano plots showing circRNA expression differences in the 3,000 m vs. 1,000 m; 1,000 m vs. sea level (0 m); and 3,000 m vs. sea level (0 m) comparisons. CircRNAs with fold change (FC) > 2 and *p* < 0.05 are colored red (upregulated) or blue (downregulated). **(C)** Heatmap of circRNA expression profiles based on hierarchical clustering analysis. Red indicates higher expression, and blue indicates lower expression.

To identify key circRNAs, we analyzed differential expression profiles obtained from the circRNA expression microarray. Scatter plots were employed to evaluate the overall expression variations in each pairwise comparison (Figure 3A), and differentially expressed circRNAs (DE-circRNAs) were identified using volcano plots (Figure 3B). A fold change (FC) > 2 and a *p*-value <0.05 were adopted as the thresholds for defining significant differential expression. The expression profiles of these DE-circRNAs were visualized through hierarchical clustering analysis (heatmap), where each row and column represents a probe set and a sample, respectively (Figure 3C). In the comparison between the 3,000 m and 1,000 m groups, 135 DE-circRNAs were identified (36 upregulated and 99 downregulated). Between the 1,000 m and sea level (0 m) groups, 20 DE-circRNAs were detected (18 upregulated and 2 downregulated). Finally, the comparison between the 3,000 m and sea level (0 m) groups yielded 41 DE-circRNAs (26 upregulated and 15 downregulated).

### Potential biological functions and pathways

**FIGURE 4:**
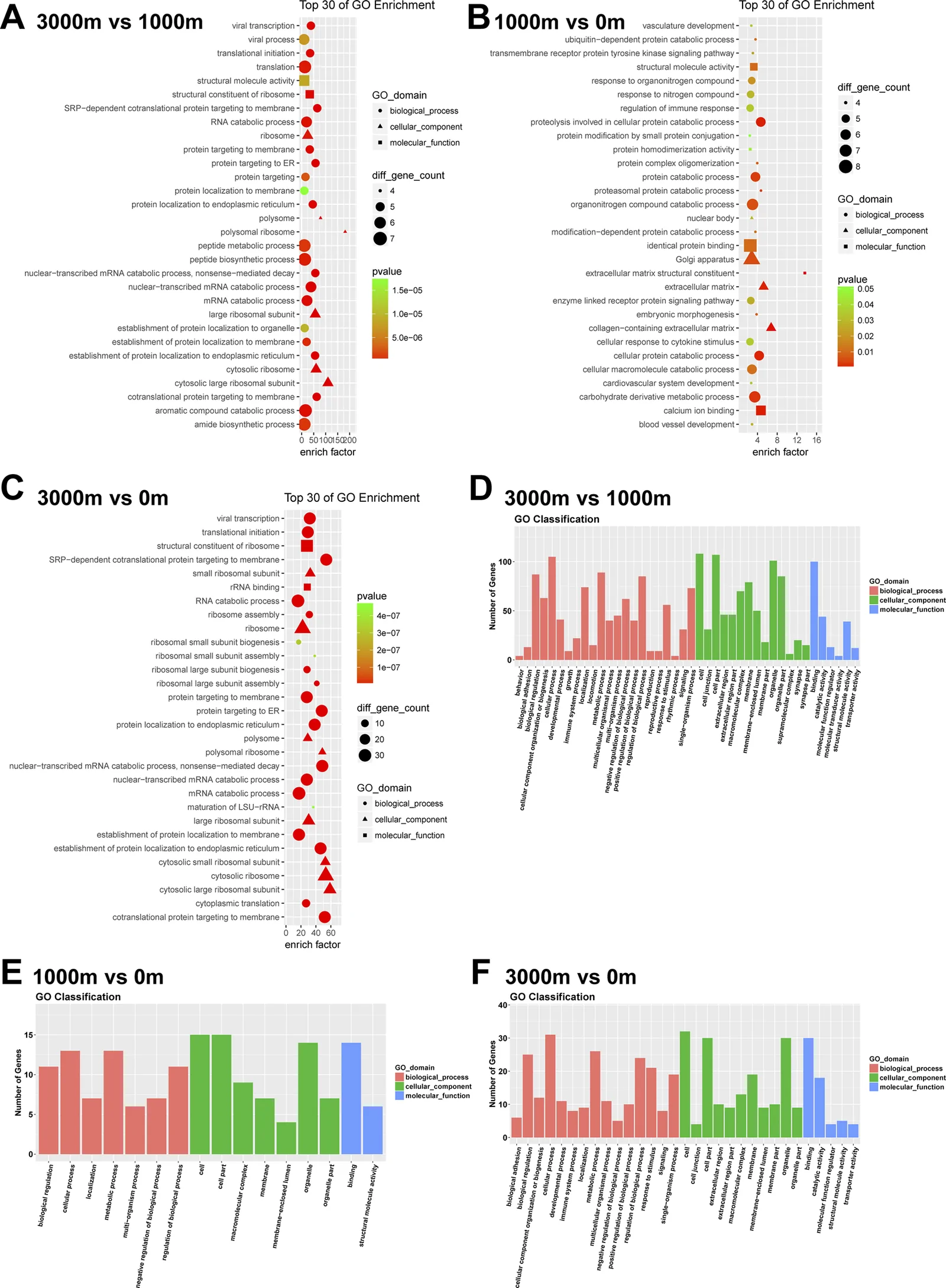
GO analysis of the parent genes of differentially expressed circRNAs. **(A)** Top 30 enriched GO terms of parent genes of differentially expressed circRNAs in the 3,000 m vs. 1,000 m comparison. **(B)** Top 30 enriched GO terms of parent genes of differentially expressed circRNAs in the 1,000 m vs. sea level (0 m) comparison. **(C)** Top 30 enriched GO terms of parent genes of differentially expressed circRNAs in the 3,000 m vs. sea level (0 m) comparison. **(D)** GO classification of parent genes of differentially expressed circRNAs in the 3,000 m vs. 1,000 m comparison. **(E)** GO classification of parent genes of differentially expressed circRNAs in the 1,000 m vs. sea level (0 m) comparison. **(F)** GO classification of parent genes of differentially expressed circRNAs in the 3,000 m vs. sea level (0 m) comparison. GO analysis was categorized into three categories: biological processes, molecular functions, and cellular components. Dot size represents the number of enriched genes, and dot color represents the statistical significance of the corresponding GO category.

To explore the potential biological functions of the differentially expressed circRNAs, we performed GO and KEGG pathway enrichment analyses. Figure 4 displays the top 30 enriched GO terms for the parent genes of circRNAs identified in each pairwise population comparison. In the 3,000 m vs. 1,000 m comparison (Figure 4A), parent genes were primarily enriched in BPs related to translational initiation, signal recognition particle (SRP)-dependent cotranslational protein targeting to membrane, and mRNA catabolic processes. CC terms were mainly associated with ribosomes and cytosolic large ribosomal subunits, while MF terms were mainly associated with structural constituents of the ribosome and rRNA binding. For the 1,000 m vs. sea level (0 m) comparison (Figure 4B), enriched BP terms included translation, peptide metabolic processes, and peptide biosynthetic processes. CC and MF terms showed similar trends to the 3,000 m vs. 1,000 m comparison, with primary enrichment in ribosomal components and structural constituents of the ribosome. In the 3,000 m vs. sea level (0 m) comparison (Figure 4C), enriched BP terms shifted toward cellular protein catabolic processes and carbohydrate derivative metabolic processes. CC terms were largely enriched in extracellular matrix components, while MF terms included structural molecule activity, identical protein binding, and calcium ion binding.

Furthermore, we performed GO functional classification analysis on the parent genes of the differentially expressed circRNAs for each pairwise population comparison (Figures 4D–F). In all three comparison groups (3,000 m vs. 1,000 m; 1,000 m vs. sea level; and 3,000 m vs. sea level), the parent genes exhibited consistent distribution patterns across GO categories. In the BP domain, the majority of genes were categorized under “cellular process,” “metabolic process,” and “regulation of biological process.” For CC terms, the genes were primarily associated with “cell,” “cell part,” and “organelle.” Within the MF domain, “binding” was the most common term across all comparisons, supplemented by “catalytic activity” and “structural molecule activity” in specific groups.

**FIGURE 5:**
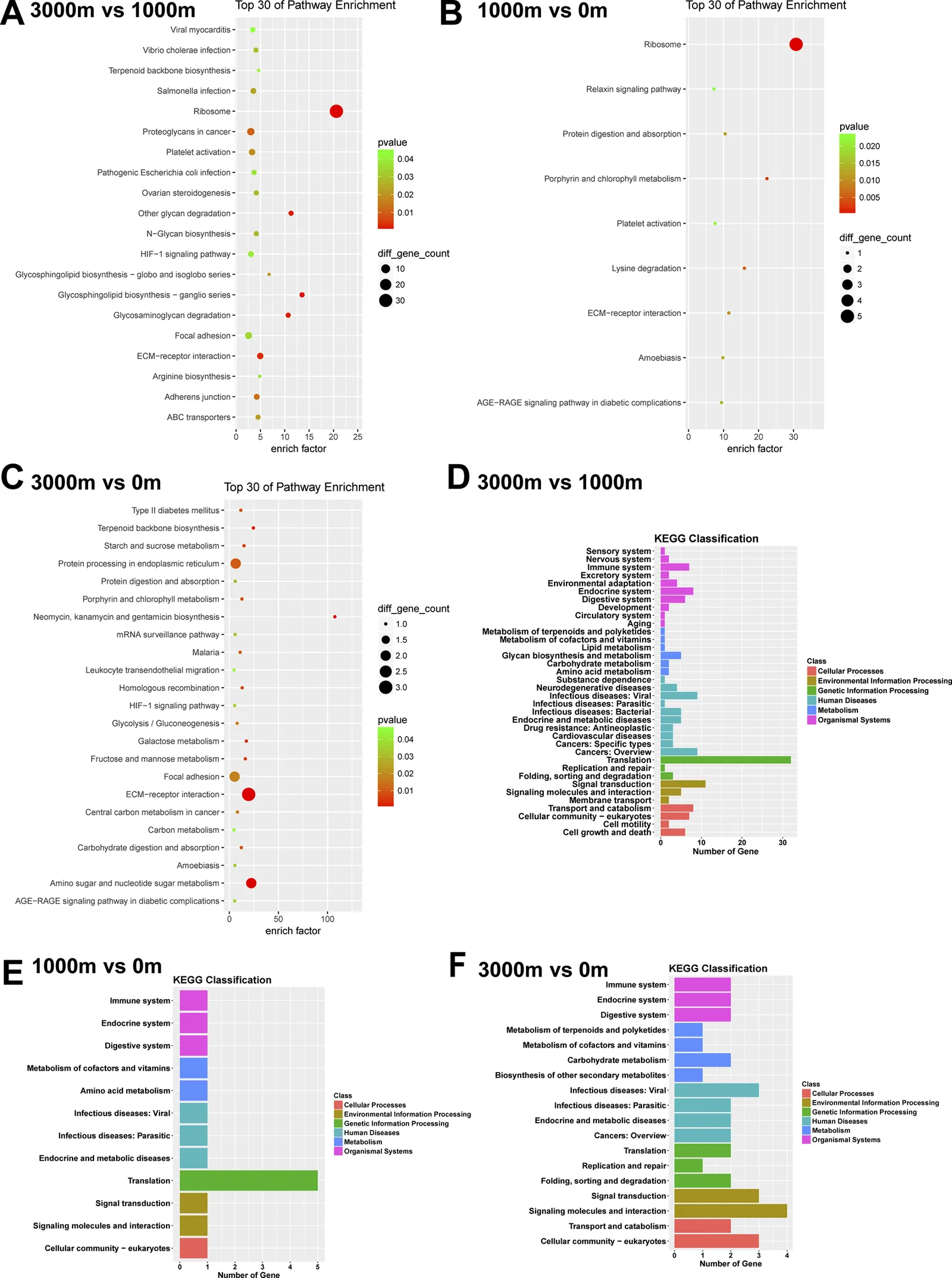
KEGG analysis of the parent genes of differentially expressed circRNAs. **(A)** Top 30 enriched KEGG pathways in the 3,000 m vs. 1,000 m comparison. **(B)** Top 30 enriched KEGG pathways in the 1,000 m vs. sea level (0 m) comparison. **(C)** Top 30 enriched KEGG pathways in the 3,000 m vs. sea level (0 m) comparison. **(D)** KEGG classification of parent genes of differentially expressed circRNAs in the 3,000 m vs. 1,000 m comparison. **(E)** KEGG classification of parent genes of differentially expressed circRNAs in the 1,000 m vs. sea level (0 m) comparison. **(F)** KEGG classification of parent genes of differentially expressed circRNAs in the 3,000 m vs. sea level (0 m) comparison. KEGG analysis was categorized into six categories: cellular processing, environmental information processing, genetic information processing, human diseases, metabolism, and organismal systems. Dot size represents the number of enriched genes, and dot color represents the statistical significance of the corresponding pathway.

To further elucidate the functional roles of the identified circRNAs, we performed KEGG pathway enrichment analysis on their parent genes. The top 30 enriched pathways for each pairwise population comparison are summarized in Figure 5. In the 3,000 m vs. 1,000 m comparison (Figure 5A), the parent genes were primarily associated with the ribosome, other glycan degradation, and ECM–receptor interaction pathways. For the 1,000 m vs. sea level (0 m) comparison (Figure 5B), the ribosomal pathway was the most prominent enrichment. Finally, in the 3,000 m vs. sea level (0 m) comparison (Figure 5C), the parent genes were mainly enriched in pathways related to protein processing in the endoplasmic reticulum and ECM–receptor interaction.

To gain further insight into the functional categories of the parent genes, we performed KEGG pathway classification analysis for each pairwise population comparison. Figures 5D–F illustrate the distribution of these genes across various KEGG functional categories. In the 3,000 m vs. 1,000 m comparison (Figure 5D) and the 1,000 m vs. sea level (0 m) comparison (Figure 5E), the parent genes were primarily associated with the “translation” category. In the 3,000 m vs. sea level (0 m) comparison (Figure 5F), the parent genes were mainly classified under “translation,” “signaling molecules and interaction,” and “signal transduction” pathways.

### circRNA analysis

**FIGURE 6:**
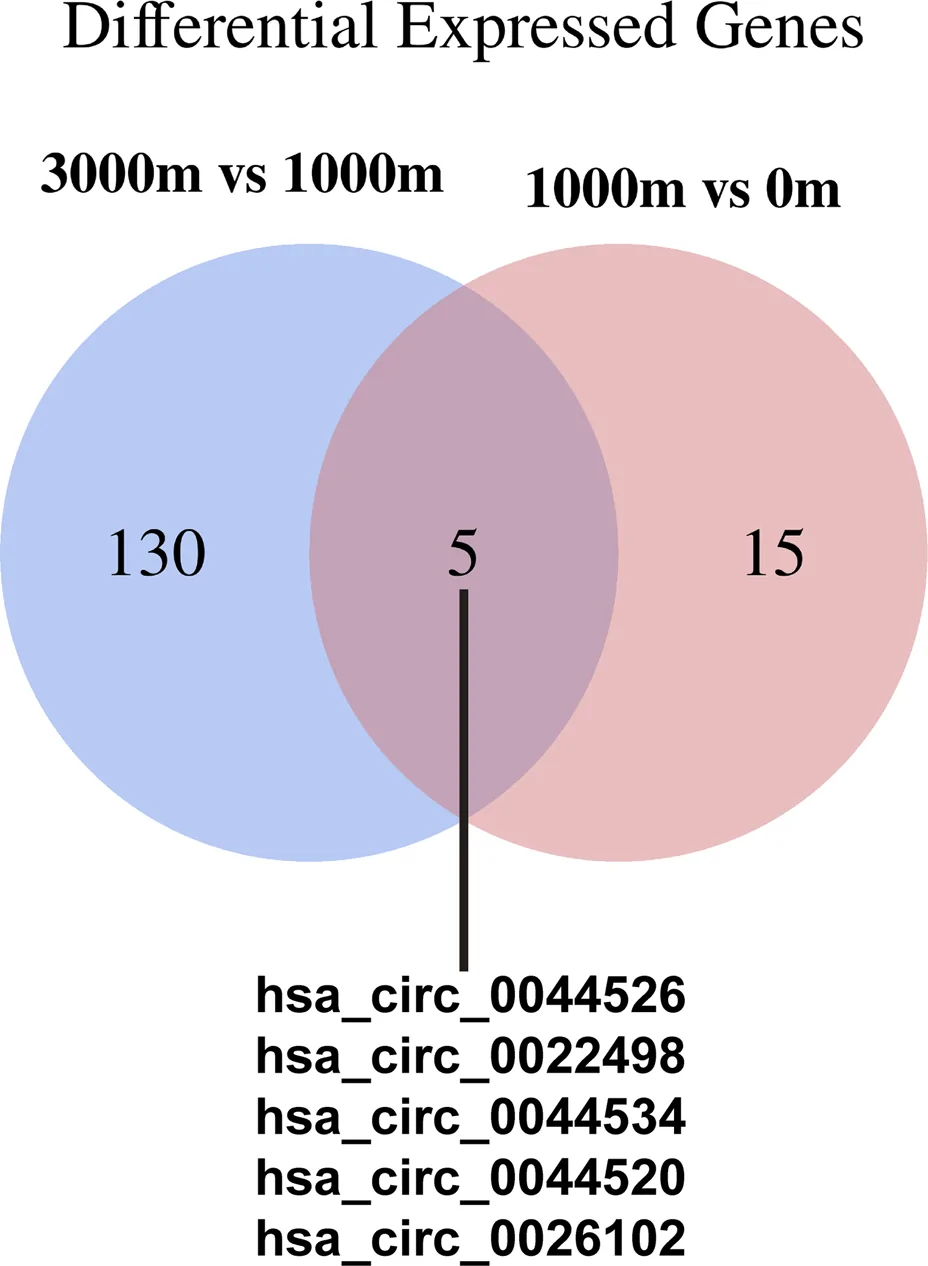
Venn diagram of differentially expressed circRNAs among the three altitude groups. A total of 135 differentially expressed circRNAs were identified between the 3,000 m and 1,000 m groups, and 20 differentially expressed circRNAs were identified between the 1,000 m and sea level (0 m) groups. Five circRNAs were shared in both comparisons: hsa_circ_0044526, hsa_circ_0022498, hsa_circ_0044534, hsa_circ_0044520, and hsa_circ_0026102.

We identified 135 differentially expressed circRNAs between the 3,000 m and 1,000 m groups, 130 of which were unique to this comparison. In the 1,000 m vs. sea level (0 m) comparison, 20 circRNAs were differentially expressed, with 15 being unique to this group. A total of five common circRNAs—hsa_circ_0044526, hsa_circ_0022498, hsa_circ_0044534, hsa_circ_0044520, and hsa_circ_0026102—were identified across both comparisons (Figure 6A). Further analysis provided detailed characteristics and predicted RNA-binding proteins (RBPs) for these five candidates (Table 1). Notably, AGO2 and EIF4A3 were identified as shared RBPs for all five circRNAs, suggesting a potential role for these candidates in post-transcriptional regulation.

**TABLE 1:** Characterization of the five key circRNAs and their corresponding RNA-binding proteins.

circRNA ID	Host gene	Genomic location	Spliced sequence length	Rbp (site count)	Database	Prediction threshold
hsa_circ_0044526	COL1A1	chr17:48,265,890–48,273,337	2,205	AGO2 (1) EIF4A3 (20)	CircBank	Score ≥0.5
hsa_circ_0044534	COL1A1	chr17:48,266,528–48,273,337	1,935	AGO2 (2) EF4A3 (22)	CircBank	Score ≥0.5
hsa_circ_0044520	COL1A1	chr17:48,264,375–48,273,337	2,529	AGO2 (3) C170RF85 (1) EIF4A (14) IGF2BP3 (1)	CircBank	Score ≥0.5
hsa_circ_0022498	UBXN1	chr11:62,443,971–62,446,527	1,289	AGO2 (1) EIF4A3 (2) HNRNPC (1)	CircBank	Score ≥0.5
hsa_circ_0026102	MLL2	chr12:49,435,871–49,448,534	5,933	AGO1 (1) AGO2 (7) DGCR8 (2) EIF4A3 (1)	CircBank	Score ≥0.5

### qRT-PCR validation

**FIGURE 7:**
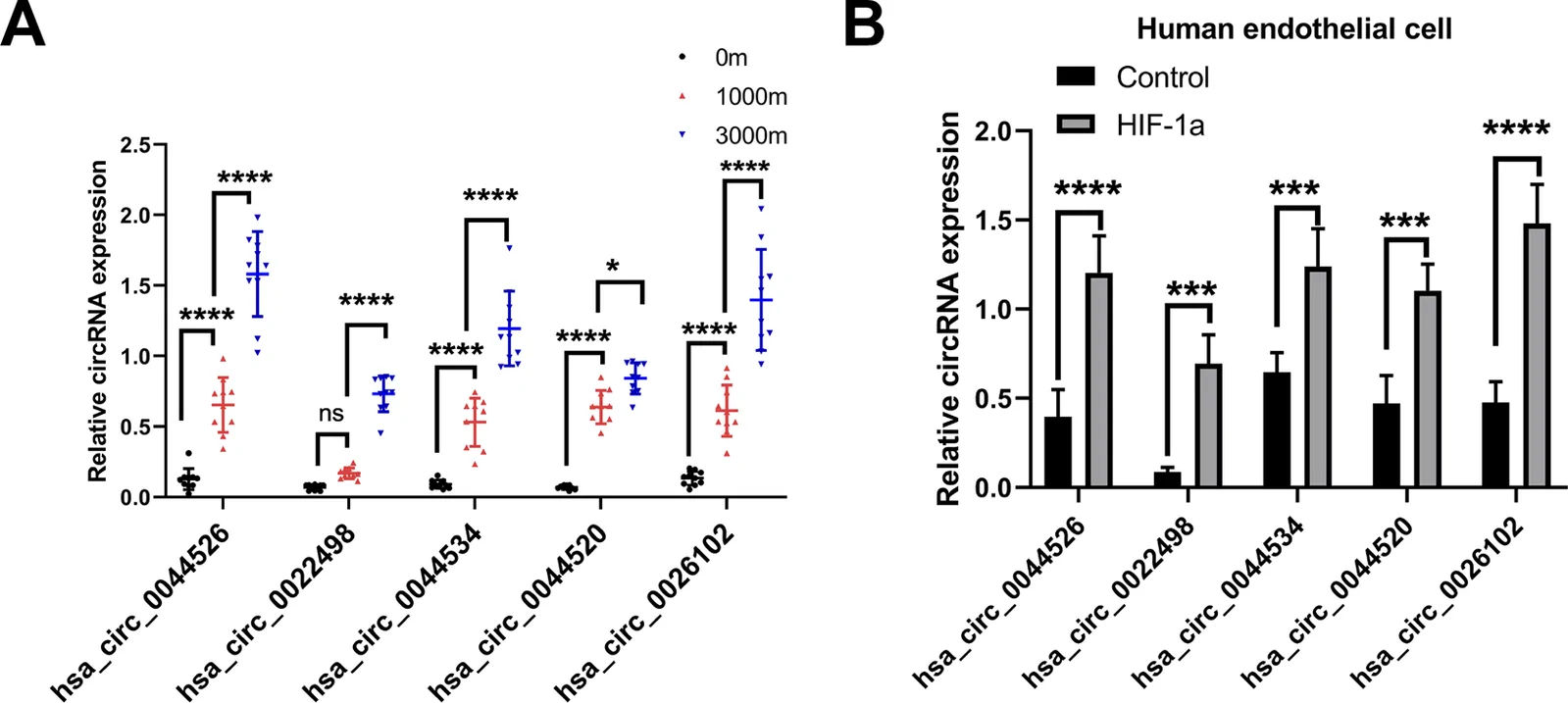
qRT-PCR verification of the microarray analysis results. **(A)** Relative expression levels of the five circRNAs were significantly upregulated with increasing altitude. Expression levels in the 1,000 m group were higher than those in the sea level (0 m) group, and expression levels in the 3,000 m group were higher than those in both the 0 m and 1,000 m groups. **(B)** Relative expression levels of the five circRNAs in HUVECs under control and HIF-1α overexpression conditions. HIF-1α overexpression significantly increased the expression levels of the five circRNAs.

To further validate the differential expression of these five circRNAs, qRT-PCR was performed. As shown in Figure 7A, the relative expression levels of these five circRNAs were higher in the 1,000 m population group and highest in the 3,000 m population group. To further confirm the results *in vitro*, the relative expression levels of these circRNAs were quantified in human endothelial cells after transfection with the HIF-1α overexpression vector. As shown in Figure 7B, HIF-1α overexpression enhanced the relative expression levels of these five circRNAs in human endothelial cells. This result reveals that the five specific circRNAs contribute to HIF-1α-mediated high-altitude adaptation.

## Discussion

Living at HA is challenging due to the low oxygen pressure. A comprehensive physiological response that protects tissues from hypoxic injury is essential for adaptation and survival (D'Alessandro and Xia, 2020). HA responses are induced by multiple factors, and HA adaptation is critical for the survival of populations. Differences in ethnicity, geographical environment, and evolutionary history may lead to different levels of HA response (Getu et al., 2025; Nieves-Colón et al., 2022; Mairbäurl et al., 2020). Therefore, it is of great significance to clarify the adaptation patterns of different populations to the HA environment. With the decreasing cost of next-generation sequencing (NGS) technology, a variety of whole-genome sequencing studies have been performed to explore the molecular mechanism of HA adaptation (Zaborowska et al., 2021; Rathore et al., 2022; Hofmann et al., 2021). A microarray study revealed that key genes associated with hypoxic stress and endoplasmic reticulum stress, including HIF-1α, STAT3, and EGFR, exhibit significantly lower expression levels in Tibetan placentas than in European placentas (Tenzing et al., 2021). In addition, a study constructed a predictive model including 50 genes, such as LRRC18 and HCAR3, by integrating two public microarray datasets, providing insights into the risk of HAPC in Han Chinese populations after exposure to high altitude (Wang et al., 2022). These studies indicate that the HA environment can extensively regulate human genome expression and that genome-wide expression detection represents an effective strategy for revealing HA adaptation mechanisms.

CircRNAs are a class of non-coding RNAs without 5′terminal caps or 3′terminal poly(A) tails, which form closed-loop structures through covalent bonding (Hansen et al., 2013; Kos et al., 1986; Wang et al., 2014). CircRNAs are mainly derived from gene exons, but other types also exist, including circRNAs originating from introns, intergenic regions, antisense strands, and sense-overlapping regions. Some circRNAs contain miRNA response elements and can act as competitive endogenous RNAs (ceRNAs) by binding miRNAs and serving as miRNA sponges in cells, thereby relieving the inhibition of target genes by miRNAs and upregulating target gene expression (Hansen et al., 2013; Shang et al., 2019). In recent years, circRNAs have been regarded as key biomarkers for many human diseases, including cancer (Conn et al., 2024; Zhang et al., 2023). However, whether circRNAs participate in HA adaptation and regulation remains unclear.

In this study, we selected permanent residents from Tianjin sea level (0 m), Ningxia Yinchuan (1,000 m), and Xizang Linzhi (3,000 m) as study subjects. All participants were healthy male individuals who had resided continuously in the corresponding area for more than 3 consecutive years and had consistent genetic backgrounds and basic lifestyles to minimize confounding factors. We performed a genome-wide expression analysis to characterize circRNA regulation in three populations living at different altitudes. As a major type of genomic structural variation, CNVs drive long-term adaptive evolution in extreme environments. Unlike SNVs with mild effects, CNVs strongly regulate gene transcription by altering gene dosage and chromatin structure. We observed CNVs in the parent genes of circRNAs and identified differentially expressed circRNAs among the three populations. Using GO and KEGG analyses, we explored the potential functions and pathways of the parent genes of differentially expressed circRNAs. It is worth noting that while functional enrichment analyses of host genes offer preliminary predictive insights, the intrinsic biological functions of circRNAs cannot be definitively established without *in vitro* experimental validation. We identified five common differentially expressed circRNAs in the three populations and predicted their binding proteins. Finally, we verified the differential expression of these five circRNAs among the three populations and confirmed by qRT-PCR that HIF-1α enhanced their relative expression. While these unidirectional transfection assays reveal a positive correlation between HIF-1α and the five circRNAs, they do not verify the reverse regulation of the HIF-1 pathway by circRNAs. In the absence of knockout and rescue experiments, this association must be interpreted as a preliminary exploratory clue rather than a definitive causal link. In summary, we identified five circRNAs differentially expressed in three populations at different altitudes: hsa_circ_0044526, hsa_circ_0022498, hsa_circ_0044534, hsa_circ_0044520, and hsa_circ_0026102. GO analysis showed that biological processes represented the most significantly enriched function of the parent genes. For instance, biological regulation was one of the most enriched terms in 3,000 m vs. 1,000 m; 1,000 m vs. sea level (0 m); and 3,000 m vs. sea level (0 m) comparisons. KEGG analysis indicated that the parent genes were involved in multiple pathways, including the HIF-1 signaling pathway, suggesting that HIF-1 may serve an important function in HA adaptation. AGO2 and EIF4A3 were the common RNA-binding proteins predicted to interact with these five circRNAs, which is consistent with previous studies reporting that AGO2 and EIF4A3 participate in hypoxic regulation (Hale et al., 2014; Li et al., 2020).

HIF-1 is a transcription factor that is essential for responses to low oxygen levels or hypoxia (Yuan et al., 2024). It consists of two subunits: the oxygen-regulated HIF-1α and the constitutively expressed HIF-1β (Wang et al., 1995; Jiang et al., 2025). HIF-1 acts as the master regulator of numerous hypoxia-induced genes under hypoxic conditions (Dengler et al., 2014). Proteins encoded by HIF-1 target genes enhance oxygen supply and mediate adaptive responses to oxygen depletion (Yang et al., 2025; Gao et al., 2025; Jin et al., 2018). Although known as a hypoxia-inducible factor, HIF-1 can induce a series of adaptive responses not only by reducing oxygen utilization but also through other stimuli, such as nitric oxide or various growth factors (Caballano-Infantes et al., 2022; Giaccia et al., 2004; So et al., 2022). In this study, KEGG analysis identified the HIF-1 signaling pathway as a key pathway for the parent genes of differentially expressed circRNAs. Moreover, we confirmed that HIF-1α increased the relative expression of the five circRNAs. This unidirectional regulatory observation provides only preliminary correlative clues linking these circRNAs to HIF-1-related hypoxic signals and altitude-associated molecular phenotypes.

This study enrolled only male subjects with a limited sample size, which may restrict the generalizability of the conclusions. The present findings are observational and correlative rather than mechanistic due to the lack of in-depth functional experiments, such as circRNA overexpression and knockdown. In addition, other vital hypoxia regulators, including EPAS1, EGLN1, and PPARA, may also contribute to high-altitude adaptation and warrant further investigation. Despite these limitations, this study provides a comprehensive overview of circRNA expression profiles in human populations at different altitudes. The identification of differentially expressed circRNAs and their association with the HIF-1 pathway expands our understanding of the genetic basis and molecular regulatory network of HA adaptation. The five circRNAs identified in this study may serve as novel molecular markers for evaluating HA adaptability. Collectively, this study reveals transcriptomic characteristics during HA adaptation and offers new insights into the molecular mechanisms underlying human adaptation to high altitude.

## Data Availability

The original contributions presented in the study are included in the article/supplementary material, further inquiries can be directed to the corresponding authors.
